# Substitute for the Mother’s Milk during Infancy

**Published:** 1878-06

**Authors:** 


SUBSTITUTE FOR THE MOTHER'S MILK DURING INFANCY.—While looking over the New Orleans *Medical and Surgical Journal* for April, 1878, my attention was drawn to an article in the "Current Medical Literature" department, entitled "Elementary Advice to Mothers and Nurses." The paper was read by M. Bienfait, before the Societe Medical de Riems, as a "draught of the advice to be given to mothers and nurses by the Society for the Protection of Childhood," was adopted by the society and published in the *Canadian Journal of Medical Science.*

While coinciding in the main with the views therein expressed, I feel compelled by experience and observation to take exception to the first article contained in the "Advice," entitled "Nursing," and to which the author has devoted nearly half the space allotted to his entire subject. Not that the rules laid down by him are essentially wrong, for if carried out to the letter, they would undoubtedly meet most of the requirements of that first and very important period of childhood. It is the almost utter impossibility of their being complied with by the great majority of people that renders the article on nursing almost a nullity as far as its usefulness is concerned. Let us pass over his prefatory remarks to the effect that "it is the duty of a mother to nourish her child from her own breast, or, her health not permitting, provide for it a nurse," as being self-evident facts, and take up that portion devoted to substitutes for human milk. Therein consists the great difficulty; and I have no doubt of the thousands of young children who die each year, and the equally great number of those that are puny and unhealthy, that one meets daily, such result is due in many instances to the unavailing attempts made to follow similar advice to that given in M. Bienfait's article.

He advocates the substitution of animal milk, that of the cow or goat, and gives minute details as to how it should be prepared, diluted, warmed, etc.; that the milk from the same animal should be used invariably, if possible, and of course the milk should be pure in the first instance.

The foregoing would prove very good, no doubt, if only within the range of common possibility. How many people resident in cities are so circumstanced as to be able to do this? Even in the country, it is physically impossible to maintain for months a regular system such as this, although pure milk is there so much more easily obtained. Indeed, one is ordinarily very fortunate if a constant supply of fresh milk obtained from different animals (of the same species) can be had. It is a known fact that the milk secreted by one and the same cow is subject to variation from day to day, said variation being controlled by the quantity, quality and variety of the food obtained, and is liable to still further changes having their origin in disturbances of the functions of digestion, from which animals are scarcely less exempt than members of the human family. Further than this, the *first* milk drawn from the cow is much thinner and less rich than the *last* or "strippings" from the same milking. Bearing this in mind, it is evident that the digestive apparatus of the child that is being fed after the fashion recommended by M. Bienfait, has to deal with a very different article of food at nearly every meal, and that, also, when of an age and development that render it eminently unfit for any such contest, the repeated renewal of which must assuredly inure to its disadvantage.

In cities, where people have to rely upon the tender mercies and elastic consciences of the professional milkman, the difficulties are augmented. If we pay for a pure article of milk, I question greatly whether it would in every instance bear thorough analysis unscathed.

Many are familiar with the anecdote of the Frenchman who sent for his milkman and bargained for a regular supply of milk at six sous per pint. He demanded that it should be pure and undiluted. "Then, monsieur, the price will be ten sous." "You must milk the cow in the presence of my valet." "In that case I will be compelled to charge fifteen sous."

The manifold difficulties in the way being sufficient to prevent the following of this advice with advantage, the question naturally arises, have we any better means at our command? Emphatically we have. I do not wish to be regarded as one who would "tear down without building again," and can say positively, basing my conclusions upon experience and repeated tests within the past four years, that in the best grade of condensed milk we have the best possible substitute for the milk of the mother.

Its advantages are many. It agrees perfectly with the child from the day of its birth; it can be freshly prepared at any moment, night or day, in any desired quantity; it is of uniform strength or richness, the contents of the cans being uniform throughout; and lastly, but not least important, the cost places it within the reach of all, and it is to be found in nearly every grocery or country store in the United States. My experience has been with the "Eagle Brand" of milk (and I would advise all to avoid the cheaper and inferior qualities), and in no single instance could any objection to its use be urged. My first experience with it was while in charge of the Obstetrical ward of the Charity Hospital, New Orleans, and the happy results obtained with it among the children born in the ward (many of whom were not nursed by their mothers at all) led me to urge it since then in my practice whenever, from any cause, artificial nursing had to be resorted to. During its preparation, the condensed milk is rendered much sweeter than that usually fed to infants, but has always, as far as my observation extended, agreed perfectly with the child. Even during dentition with accompanying diarrhœa, so often seen in practice, it has never produced any exaggeration of the disease in any case I have met with.

At the present time I have under my observation a male child seven months old, that has been fed *exclusively with condensed milk;* having been denied all other food or drink since birth. It is very robust, and has not had a day's sickness; is now teething without trouble. At its birth it weighed seven and one-half pounds, when six months old weighed twenty-one and one-half pounds, and has increased in weight very perceptibly within the past month: a fair specimen of a fat, muscular little christian, and an equally good one of the effects of an exclusive diet of condensed milk. A lady in my vicinity having lost two children consecutively while teething, from general debility and mal-nutrition, brought about by an insufficient supply of her own milk and the wretched substitute therefor, namely, that of the cow, with all its accompanying ills, is now using this preparation for her third child with good results, and bids fair to succeed in raising it with but little trouble.

The method of giving the condensed milk is very simple. In the first month dissolve a dessert-spoonful in about three-fourths of a pint of hot water, and then let it cool to the temperature of the body. Each successive month slightly increase the quantity of milk until the sixth, when the maximum is reached of two large table-spoonfuls to the pint of milk.

As regards the hours for feeding, given with such precision by M. Bienfait, I have only to say that in permitting the instinct of the child to be the guide, one will seldom go astray. When it is hungry it very generally makes it manifest, and if not neglected for too long a period, will never take more than it actually needs. The best nursing bottle I find to be a common six or eight ounce, clear glass bottle, provided with a plain black rubber nipple. Each time it is used, both the bottle and nipple should be thoroughly washed, and the latter placed in a glass of cold water until again wanted. The bottles and black rubber nipples are both very cheap, and to be had from any druggist. The regulation nursing bottle (as usually made, with fancy cap, rubber tube, etc.) is more expensive, and very difficult to keep thoroughly clean.

In nearly every instance, I have had to overcome a prejudice in the minds of the parents against what they considered an artificial milk, but have subsequently been gratified by their hearty endorsement.

Should I—by presenting thus publicly this easy and simple mode of properly nourishing children when the maternal source fails them—be instrumental in ever so slight a degree in ameliorating the condition or saving the life of only one little innocent, I shall feel that my labor has not been in vain.—*G. A. B. Hays, in the New Orleans Medical and Surgical Journal.*

